# Temperature Stability and Spectral Tuning of Long Period Fiber Gratings Fabricated by Femtosecond Laser Direct Writing

**DOI:** 10.3390/s20143898

**Published:** 2020-07-13

**Authors:** Duarte Viveiros, José M. M. M. de Almeida, Luís Coelho, Helena Vasconcelos, João M. Maia, Vítor A. Amorim, Pedro A. S. Jorge, Paulo V. S. Marques

**Affiliations:** 1 Center for Applied Photonics, INESC TEC, 4200-465 Porto, Portugal; jmmma@utad.pt (J.M.M.M.d.A.); lcoelho@inesctec.pt (L.C.); helena.s.vasconcelos@inesctec.pt (H.V.); joao.m.maia@inesctec.pt (J.M.M.); vitor.a.amorim@inesctec.pt (V.A.A.); pedro.jorge@fc.up.pt (P.A.S.J.); psmarque@fc.up.pt (P.V.S.M.); 2 Department of Physics and Astronomy, Faculty of Science, University of Porto, 4169-007 Porto, Portugal; 3 Department of Physics, University of Trás-os-Montes e Alto Douro, 5001-801 Vila Real, Portugal

**Keywords:** femtosecond laser, long period fiber gratings, optical fiber sensors, temperature, thermal treatment, turn around point

## Abstract

Long period fiber gratings (LPFGs) were fabricated in a standard single mode fiber (SMF-28e) through femtosecond (fs) laser direct writing. LPFGs with longer and shorter periods were fabricated, which allows coupling from the fundamental core mode to lower and higher order asymmetric cladding modes (LP_1,6_ and LP_1,12_, respectively). For the grating periods of 182.7 and 192.5 µm, it was verified that the LP_1,12_ mode exhibits a TAP at approximately 1380 and 1448 nm in air and water, respectively. Characterization of the LPFGs subjected to high-temperature thermal treatment was accomplished. Fine-tuning of the resonance band’s position and thermal stability up to 600 °C was shown. The temperature sensitivity was characterized for the gratings with different periods and for different temperature ranges. A maximum sensitivity of −180.73, and 179.29 pm/°C was obtained for the two resonances of the 182.7 µm TAP LPFG, in the range between 250 and 600 °C.

## 1. Introduction

Long period fiber gratings (LPFGs) are applied to couple light between the core mode and the co-propagating cladding modes in different types of fibers [1], such as photonic crystal fibers [2], standard single mode optical fibers [3], polarization-maintaining fibers [4], and photosensitive fibers [5,6]. They are intrinsically sensitive to external perturbations, enabling the measurement of several parameters [7]. The fabrication of an LPFG with high sensitivity to the external perturbations is possible by selecting a cladding mode and period at, or very close to, the turning around point (TAP) [8,9,10,11,12,13].

Several methods have been explored for the fabrication of LPFGs, including exposure to UV and CO_2_ laser radiation [14,15,16,17], electric arc discharge [18,19], and mechanical pressure [1]. However, post-processing techniques such as taper tuning [10], cladding etching [20], and overlay coating [10,11] are necessary for the fabrication and tuning of an LPFG at the TAP. These fabrication processes can weaken the optical fiber and render its implementation difficult [1].

The femtosecond (fs) laser direct writing technique opens the possibility to inscribe LPFGs inside the core volume with high spatial resolution due to the underlying non-linear light–matter interaction [8,21]. This technique shows excellent design flexibility and enables rapid change of the LPFGs parameters, such as length, bandwidth, or period, unlike other fabrication methods [18,22,23,24].

The LPFGs fabrication in standard SMF-28e fiber employing a fs-laser system and its sensing characteristics to temperature, external refractive index (RI), and strain have already been demonstrated [3,7,23,24]. However, fs-laser LPFGs inscription in SMF-28e at the TAP has not been studied systematically and extensively.

Temperature sensitivity of 124 pm/°C at temperatures above 450 °C were reported [3]. However, the experiments reported so far only characterize the LPFGs thermal response and thermal stability and do not describe in detail the spectral behavior of the LPFGs at room temperature after exposing it to a certain temperature. Temperature-induced spectral changes can be used to tune the resonant wavelength and to improve thermal sensitivity.

A TAP LPFG having a single attenuation band in air, when immersed in a medium having a higher RI, or annealed at high temperature, causes the single resonance band to split into two resonances [1]. Consequently, the sensitivity of the TAP LPFGs is reduced. Therefore, properly addressing issues related to the TAP LPFG fabrication, such as the exact period to ensure the operation at the TAP and, at the same time, to have thermal stability at higher temperatures, is fundamental to maintain the high sensitivity characteristics [13].

In this work, the entire fabrication and characterization process of the LPFGs in SMF-28e fibers inscribed by fs-laser direct writing with periods to couple to low and high order cladding modes is described. The cladding modes were identified through near-field intensity distribution measurements. The choice of the fabrication parameters in order to obtain an LPFG in the TAP condition is also discussed. Then, the LPFGs thermal annealing was realized to improve temperature stability.

## 2. LPFGs Theory

LPFGs consist of a periodic modulation of the RI along the longitudinal axis of an optical fiber with periods greater than 100 µm, which couple light from the core-guided mode (LP_01_ mode) into forward-propagating cladding-guided modes (LP_l,m_, linearly polarized modes with azimuthal and radial integer indices *l* and *m*, respectively) at or near-resonant wavelengths [14,25]. The interaction between the core mode and the co-propagating cladding modes, propagation constants $\beta_{0,1}$ and $\beta_{l,m}$ respectively, is described by [14,25]:

(1) $$\Delta \beta \;=\;\beta_{0,1}\;-\;\beta_{l,m}\;-\;\frac{2\pi }{\Lambda }\;,\;\beta_{0,1}\;=\;\left(\frac{2\pi }{\lambda }\right)n_{core}^{eff}\;,\;\beta_{l,m}\;=\;\left(\frac{2\pi }{\lambda }\right)n_{cl\;l,m}^{eff}$$

where Δβ is the detuning factor, and Λ is the grating period. Under the phase-matching condition (Δβ =0), the resonant wavelength $\lambda_{res}$ of an LPFG is determined by [1,13,25,26]:

(2) $$\lambda_{res}\;=\;\left(n_{core}^{eff}\left(\lambda \right)-n_{cl\;l,m}^{eff}\left(\lambda \right)\right)\Lambda$$

with $n_{core}^{eff}$ as the effective RI of the propagating core mode at wavelength λ, and $n_{cl\;l,m}^{eff}$ as the effective RI of the $m^{th}$ cladding mode, respectively. Any change in the surrounding RI alters the effective indices of the cladding modes inducing changes in the wavelength position and the attenuation intensity at the resonance band. The grating’s transmission spectrum is characterized by a set of transmission dips, corresponding to different cladding modes, at wavelengths that satisfy Equation (2). Consequently, the expression to calculate the minimum transmission of the attenuation bands is defined as [1,25,26]:

(3) $$T_{m}\;=\;1-\sin^{2}\left(k_{m}L\right)$$

where $k_{m}$ is the coupling coefficient for the $m^{th}$ cladding mode, and L is the length of the LPFG. The coupling coefficient is determined by the overlap integral of the core and cladding mode and by the amplitude of the periodic modulation of the mode propagation constants. The wavelength dependence is determined by the material and waveguide dispersion [25,26]. The material dispersion can be assumed to have the same overall effect on $n_{core}^{eff}\left(\lambda \right)$, as well as $n_{cl\;l,m}^{eff}\left(\lambda \right)$ [25]. Therefore, the waveguide dispersion is the dominant contributor to the grating spectra evolution and is given by [25]:

(4) $$\gamma \;=\;\frac{\frac{d\lambda_{res}}{d\Lambda }}{\left(n_{core}^{eff}\left(\lambda \right)\;-\;n_{cl\;l,m}^{eff}\left(\lambda \right)\right)}$$

**Figure 1:**
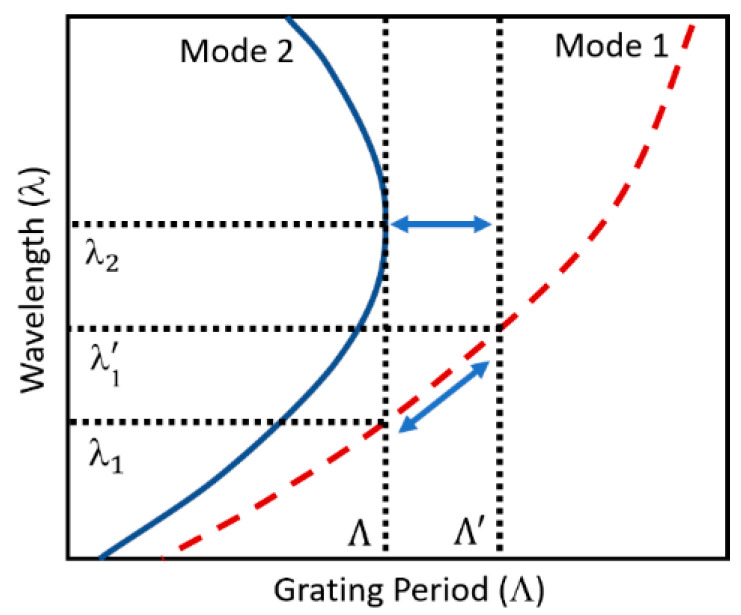
Sketch of the phase-matching curves.

The central wavelengths of the LPFGs attenuation bands are determined through the calculation of the RI of the core and cladding modes. The coupling strength and the form of the LPFGs spectrum are achieved with the calculation of the electric field mode profiles [25]. The effective RI of the propagating core mode of the fiber is generally determined using the weakly guided field approximation [1]. A sketch of the phase-matching curves of mode resonance wavelength against the grating period of an LPFG, is shown in Figure 1.

The two phase-matching curves, shown in Figure 1, present different slope values according to the LPFG order mode. The slope value of the phase-matching curves increases from low- to high-order modes, and each one reaches a maximum at a specific TAP where $\left|d\lambda_{res}/d\Lambda \right|\to \infty$ [27]. The TAP occurs at longer wavelengths for lower order modes, shifting to shorter wavelengths as the mode order increases [13].The fabrication of LPFGs with longer periods allows the coupling to lower-order modes (e.g., mode 1), while shorter periods facilitate coupling to the higher-order cladding modes (e.g., mode 2). According to the grating period, three types of LPFGs can be distinguished: positive linear-dispersion gratings for $\frac{d\lambda_{res}}{d\Lambda }>0$ $(\lambda \;<\;\lambda_{2}),$ negative linear-dispersion gratings for $\frac{d\lambda_{res}}{d\Lambda }<0$ $(\lambda \;>\;\lambda_{2}),$ and quadratic-dispersion grating for $\frac{d\lambda_{res}}{d\Lambda }=0$ $\left(\lambda \;\approx \;\lambda_{2}\right)$ [27].

A resonance with positive linear dispersion is presented by the coupling between the core mode and low-order cladding mode 1. A monotonic relationship between the LPFG period and the resonant wavelength occurs when the resonance shifts from $\lambda_{1}$ to $\lambda_{1}^{’}$ as the grating period shifts from Λ to Λ’. The turning point in the cladding mode 2 phase-matching curve occurs for a specific wavelength $\lambda_{2}$, and any deviation toward smaller periods gives origin to two attenuation bands. The high derivative around the turning point is what makes the TAP gratings very attractive.

The LPFGs fabricated with periods higher than Λ result in different coupling conditions, which makes the resonance vanish. The coupling between the core and cladding mode 2 changes its character according to Equation (1). Consequently, for increasing λ, it requires a substantial decrease of the cladding mode’s index (especially pronounced for high-order cladding modes), resulting in a positive difference in the effective indices. However, a negative difference in the effective indices switches the sign of the slope of the phase-matching curve. The quadratic dispersion case occurs when the difference in the effective indices is exactly balanced. Therefore, at each turning point, $\left|d\lambda_{res}/d\Lambda \right|\to \infty$, which results in a waveguide dispersion value of |γ|→∞. As a result, for each cladding mode, the maximum sensitivity is determined by the turning point [27]. Thus, LPFGs with high sensitivity can be fabricated by selecting a cladding mode and a period that allows operating at, or very close to, a turning around point [13].

### Temperature Sensitivity

The period of the LPFGs, the order of the cladding mode, and the composition of the optical fiber influence the sensitivity of LPFGs to temperature [1]. As demonstrated in [1], the LPFGs temperature sensitivity can be determined by differentiating Equation (2):

(5) $$\frac{d\lambda^{m}}{dT}\;=\;\frac{d\lambda^{m}}{d\left(\Delta n_{eff}\right)}\;\frac{d\left(\Delta n_{eff}\right)}{dT}\;+\;\Lambda \frac{d\lambda^{m}}{d\Lambda }\cdot \frac{1}{L}\frac{dL}{dT}$$

where *T* is the temperature, $\Delta n_{eff}$ is the difference in the effective indices ($\Delta n_{eff}\;=\;n_{core}^{eff}\;-\;n_{cl\;l,m}^{eff}$), and *L* is the length of the LPFG. The first term on the right-hand side (RHS) of Equation (5) represents the material contribution. This is related to the change in the different RI of the core and cladding arising from the thermo-optic effect. The material contribution is strongly dependent upon the order of the cladding mode and also dependent on the fiber composition. The second term on the RHS of Equation (5) represents the waveguide contribution, which results from changes in the LPFGs period and depends on the thermal expansion coefficient of the fiber [1].

## 3. Materials and Methods

### 3.1. Fabrication Procedures

**Figure 2:**
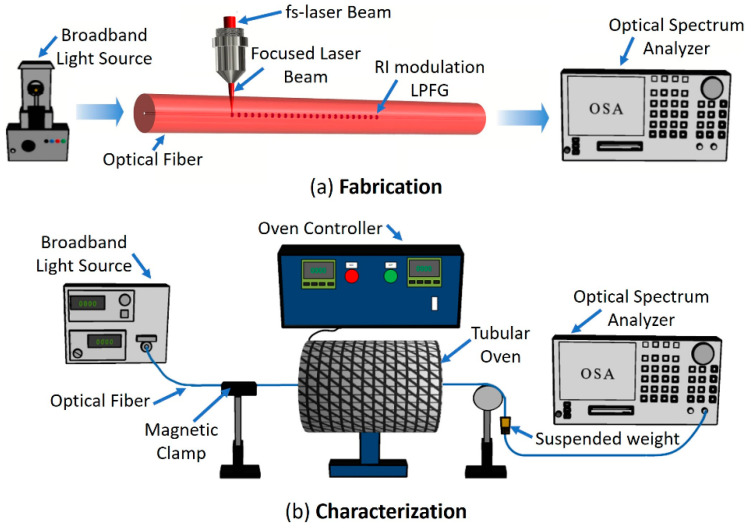
Schematic illustration of the long period fiber gratings (LPFGs): (**a**) fabrication, and (**b**) characterization procedures.

The fabrication of LPFGs inside the SMF-28e Corning optical fiber was realized through a home-assembled laser direct writing system. The system uses the second harmonic (515 nm) of a fiber amplified fs laser (Satsuma HP, from Amplitude Systèmes) with a pulse duration of approximately 250 fs and with the pulse repetition rate set to 500 kHz. The laser beam was focused inside the fiber core with a 40× aspherical lens (Newport 5722-A-H) with a numerical aperture of NA = 0.5, as illustrated in Figure 2a. The polarization of the linear polarized writing beam was set parallel to the scanning direction. The writing lens was mounted in Y (Newport (M-)ILS150CC) and Z (Newport VP-25XA) precision linear stages, which allows precise alignment of the lens over the fiber. The lens was static during fabrication, and the optical fiber was scanned along the X-direction (which is parallel to the fiber axis) by the movement of a high-quality air-bearing linear stage (Aerotech ABL10100-LN) in which the fiber was mounted [21].

Before writing, the coating was removed, the fiber was cleaned, and then, it was held in position by two magnetic clamps. Each clamp is part of a 3-axis positioner that is mounted in the X-stage. The precise alignment of the fiber core is realized using the charge-coupled device (CCD) camera image acquired through the writing lens. A calibrated load cell (Interface SML series) was assembled in the fiber holder to allow adjustment and real-time monitoring of the fiber tension. The optical fiber was kept under tension (1 N) during fabrication. A function generator (DS345, Stanford Research Systems) was utilized to generate a periodic square function with a duty cycle of 50% applied to the laser gate, determining the laser on/off frequency, while the optical fiber is translated at a constant velocity (v) along X [21]. In summary, the LPFGs were fabricated with a pulse energy of 130 nJ, 50 μm/s scan velocity, fiber tension of 1 N, writing beam polarization aligned with the scanning direction, and with different lengths and modulation periods (depending on the resonance wavelength desired).

### 3.2. Characterization Procedures

The spectral characteristics of the LPFGs were monitored in real-time during writing, by coupling broadband light (from a halogen lamp) to the fiber, while the other end is connected to an Optical Spectrum Analyzer (OSA), model ANDO AQ-6315B. The signal was measured without polarization control from 1000 to 1700 nm with a 10 nm resolution. All spectra were normalized to the transmitted spectrum of the light source.

In order to assess the symmetry of the perturbations in the fiber, the near fields of several cladding modes excited by the fs laser gratings inscription were observed. One end of the fiber was coupled to a tunable laser (Santec TSL-210V), allowing wavelength scanning around the grating resonance, while the other end was cleaved just after the grating. The near-field profile of the radiation emerging from the fiber was detected by an infrared camera (charge-coupled device, CCD, model: Point Grey Research CMLN-13S2M-CS). A 4× microscope objective was used to image the fiber cross-section at the grating end into the infrared camera.

A temperature characterization setup was mounted to measure the LPFGs spectrum at different surrounding mediums and temperatures. The setup consisted of a sliding tubular oven (Termolab), an acrylic chamber filled with water mounted on three-axis positioners (MBT616/M, Thorlabs), and an OSA (model Yokogawa AQ6370D). The optical fibers with the LPFGs were introduced in a horizontal tubular oven, being subjected to an axial tension produced by a suspended weight of 5.8 g, as illustrated in Figure 2b. The LPFGs spectra were recorded 5 min after the target temperature was reached. Then, the fiber was withdrawn from the oven after each temperature step, let cool to room temperature, and then the spectra was measured again with the fiber in air and immersed in water (the latter was only done for the TAP LPFGs). Then, the temperature was increased, and the LPFGs were introduced again in the oven. This procedure was repeated for a temperature in the range of 100 to 600 °C in steps of 50 °C. The described thermal treatment was performed two times for the regular LPFG and once for the TAP LPFGs.

Depending on the LPFGs under test, two light sources and an OSA were utilized to optimize the spectrum measurement. Thus, an amplified spontaneous emission (ASE) broadband light source (model ASE2000) was used for measurements from 1450 to 1650 nm with a 1 nm resolution and a halogen lamp for measurements from 1000 to 1700 nm with a 2 nm resolution.

## 4. Results

### 4.1. Characterization of the LPFGs Writing

**Figure 3:**
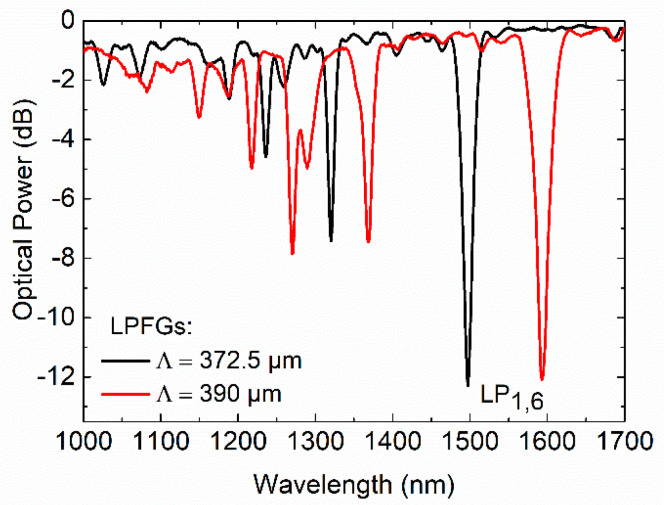
Spectra of fs-laser direct written LPFGs in single mode fiber (SMF-28e).

Following the procedures described in the fabrication section, two LPFGs were first fabricated with different RI modulation periods (372.5 and 390 µm), 30.0 and 28.5 mm long, respectively. The transmission spectra are shown in Figure 3.

As can be seen in Figure 3, different periods with the same fabrication conditions lead to resonances at specific wavelengths; the position of the attenuation band shifts toward longer wavelengths (Δλ = 95.64 nm) as the grating period increases from 372.5 to 390 µm. Furthermore, the grating strength increases, and the bandwidth decreases with the increase of the writing length.

**Figure 4:**
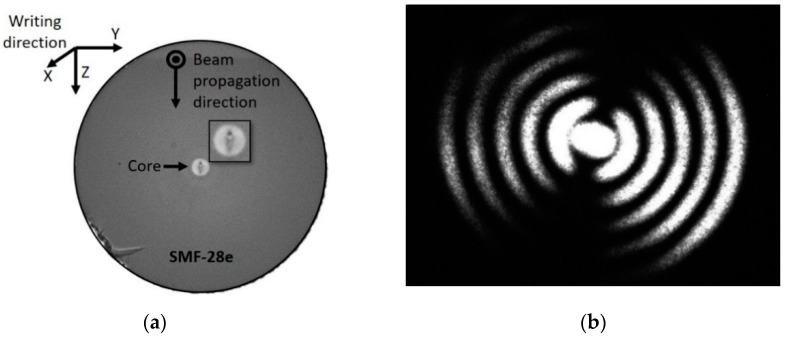
Fs laser inscribed LPFG in the SMF-28e fiber with a 8.2 µm core: (**a**) Optical microscope view of the cross-section; (**b**) Near-field intensity distribution of the cladding mode LP_1,6_ at 1592.7 nm (grating period of 390 µm).

To assess the symmetry of the perturbation of a 390 µm fs laser inscribed LPFG, the optical fiber was cleaved, and the cross-section of the LPFG structure was inspected, with an optical microscope. Figure 4 shows a picture of the structure taken by an optical microscope (a) and the corresponding near-field intensity distribution acquired (b).

The fs laser-induced RI modification presents a transversal inhomogeneous, asymmetric, elliptic shape that extends across the core center, as shown in the inset of Figure 4a. Thereby, in the SMF-28e, light is coupled to asymmetric LP_1,j_ cladding modes of order j. Accordingly, the coupling strength is strongly influenced by the structure of the modified cross-section, including non-uniformity and localization. The intensity distribution presented in Figure 4b shows that for a period of 390 µm, coupling occurs to the lower order asymmetric LP_1,6_ cladding mode. Due to the asymmetrical shape of the RI modification, for shorter periods (<190 µm), coupling also occurs to asymmetric modes. For LPFGs written with short periods, the cladding mode identification was not possible through the near-field intensity profile, because the resonances were out of the laser tuning range. The phase-matching curves for the LPFGs written in the SMF-28e fiber coupling to asymmetric cladding modes were already reported [19]. These simulated phase-matching curves do not fit precisely with the fs-laser gratings fabrication parameters. However, they can be used to claim that for a period around 187 ± 5 µm, coupling occurs to the higher order asymmetric LP_1,12_ cladding mode.

**Figure 5:**
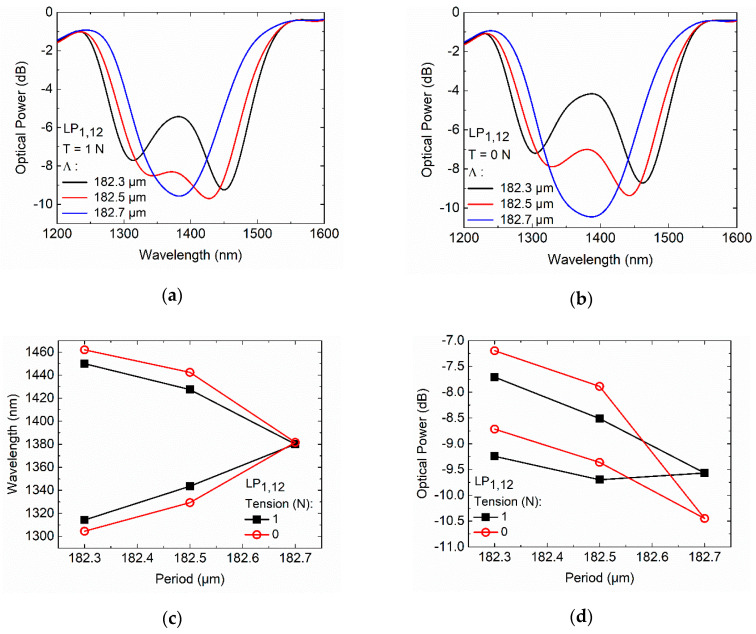
Fs laser turning around point (TAP) LPFGs higher order asymmetric cladding mode (LP_1,12_), inscribed in the SMF-28e fiber: (**a**) spectra under fabrication tension of 1 N; (**b**) spectra without tension; (**c**) resonance wavelengths versus grating period; (**d**) optical power versus grating period.

LPFGs with shorter periods (182.3, 182.5, and 182.7 µm) were also fabricated to study the behavior at, or very close to, a TAP in air. The length of the LPFGs was 19.0 mm. The transmission spectra in air, with and without applied tension, are shown in Figure 5.

The transmission spectra presented in Figure 5a exhibit two peaks (fiber surrounded by air and under tension) for periods of 182.3 and 182.5 µm. As can be seen in Figure 5b, relaxing the optical fiber increases the separation of the peaks, reaching a separation of 158.50 nm (blue-shift −10.75 nm, and red-shift +12.00 nm) and 113.25 nm (blue-shift −14.25 nm, and red-shift +15.00 nm) for periods of 182.3 and 182.5 µm, respectively. Furthermore, the intensity of both resonances of each LPFG diminishes. It can also be seen that the increase of the inscription period to 182.7 µm causes the two resonance bands in the transmission spectrum to merge into one band, i.e., the 182.7 µm period coincides with the peak of the dispersion curve of the LP_1,12_ cladding mode. Thus, the LP_1,12_ cladding mode exhibits a turning point for a wavelength around 1380 nm, as shown in Figure 5c. The optical fiber relaxation with the 182.7 µm TAP LPFG results in a change in the coupling efficiency, but not in the wavelength, as shown in Figure 5c,d. The wavelength and intensity changes of the LPFGs resonance bands with the optical fiber relaxation are associated with the Poisson’s effect (change in transverse dimensions), and to the waveguide contributions, which depend on the slope of the characteristic curve of the resonance band [11].

Several LPFGs were fabricated in order to optimize and assess the reproducibility of the fs laser direct writing. Table 1 presents a comparison between two pairs of LPFGs inscribed with periods of 372.5 and 182.7 µm, respectively.

As can be observed, a resonance wavelength difference of 0.07 nm, an intensity variation of 0.15 dB, and a full width at half maximum (FWHM) difference of 0.66 nm were achieved for the LPFGs fabricated with a period of 372.5 µm. For the TAP LPFGs fabricated with a period of 182.7 µm, we verified a resonance wavelength difference of 0.02 nm, an intensity variation of 1.41 dB, and an FWHM difference of 1.01 nm. To obtain gratings operating at the TAP, the sub-micron period control is of utmost importance, as well as the fiber tension during fabrication. The TAP LPFG intensity reproducibility is the most challenging parameter to achieve, but for the application reported here, this parameter was not critical.

Considering the wide range of applications of LPFGs based on RI sensing in aqueous solutions, an LPFG with 192.5 µm was fabricated to operate at TAP when immersed in water. The fabrication period was achieved by monitoring the LPFG spectral behavior immersed in water after the fabrication. The fabrication pulse energy and grating length were also adjusted to 120 nJ and 40 mm, respectively, in order to improve the coupling strength. Thus, the LP_1,12_ mode exhibits a TAP at approximately 1448 nm in water.

The coupling behavior changes because the surrounding refractive index increased, which induced a re-distribution of the cladding modes. According to Equation (1), the grating period increase balances the increase of the cladding mode’s index, which leads to the quadratic dispersion case in water.

### 4.2. Temperature Characterization

#### 4.2.1. Long Period Fiber Gratings

**Figure 6:**
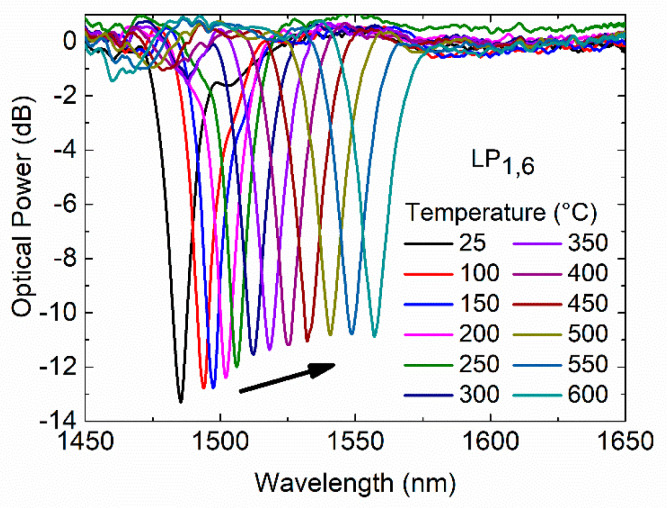
Transmission spectra of the cladding mode LP_1,6_ of a 372.5 µm LPFG at annealing temperature.

During the annealing procedure, the transmission spectra of the cladding mode LP_1,6_ of a 372.5 µm LPFG were recorded and are shown in Figure 6.

As the temperature increases from 25 to 600 °C in steps of 50 °C, the resonance wavelength is gradually red-shifted, and the intensity loss decreases. The LPFG temperature response was recorded for two annealing cycles at the annealing and room temperature.

**Figure 7:**
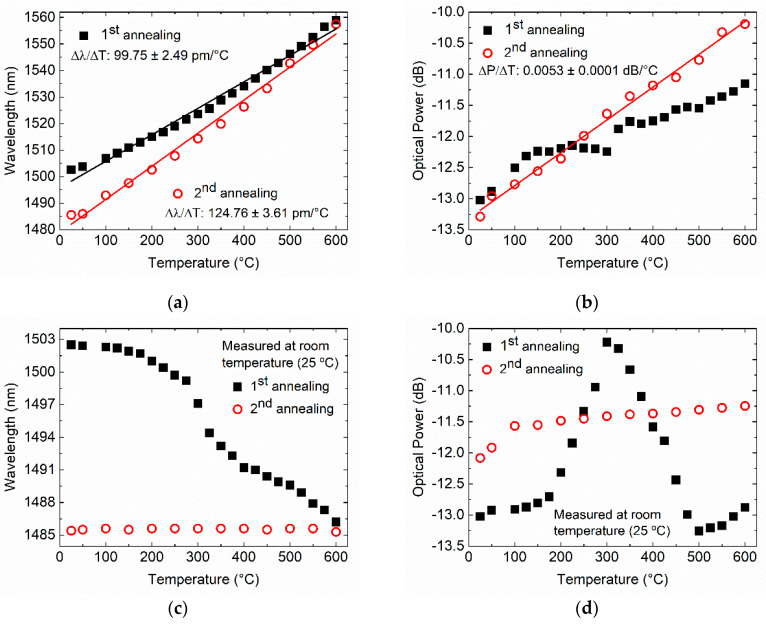
Fs-laser LPFG inscribed with a period of 372.5 µm: (**a**) wavelength shift, and (**b**) strength of the peak attenuation at the annealing temperature; (**c**) wavelength shift, and (**d**) strength of the peak attenuation, at room temperature.

Figure 7a shows for the first annealing cycle, a wavelength shift proportional to the temperature increase from 25 to 600 °C, at a rate of 99.75 ± 2.49 pm/°C (and $R^{2}\;>$ 0.99). The resonance optical power decreases non-uniformly with the annealing temperature, as shown in Figure 7b. The behavior of the LPFG was also recorded at room temperature after a dwell time of 5 min at each annealing temperature. A resonant wavelength blue-shift from 1502.51 to 1486.21 nm (Δλ = 16.30 nm) was observed at the end of the first annealing, as shown in Figure 7c. As can be seen from Figure 7d, for the first annealing, the optical power decreased for the range from 100 to 300 °C, and then it increased up to 500 °C. The optical power decreased again between 500 and 600 °C. The shift of the resonance to smaller wavelengths at the end of the annealing was mainly due to the change of the effective RI of the core mode [28]. This is because thermal activation defects caused by the fs laser inscription are annealed out, leaving a smooth and uniform RI modification between the structurally modified region and the unaffected region inside the core of the SMF-28e fiber [24,29,30].Accordingly, the LPFG exhibits poor thermal stability in their optical power because of the Type I RI changes produced by the fs laser LPFG inscription [30].

The second annealing shows an improvement of the linearity of the wavelength temperature dependence with 124.76 ± 3.61 pm/°C (and $R^{2}>$ 0.99). Furthermore, the resonance optical power decreases uniformly with the annealing temperature, presenting a temperature dependence of 0.0053 ± 0.0001 dB/°C with linearity $R^{2}>$ 0.99, as shown in Figure 7b. At room temperature (25 °C), the resonance wavelength became essentially constant at 1485.54 nm, regardless of the annealing temperature. Concerning the resonance optical power, we observed a decrease of around 0.52 dB up to 100 °C, followed by a reduction of 0.3 dB up to 600 °C. Thus, with the performed thermal treatment through short-term thermal exposure (5 min at each temperature), it is possible to enhance the fs laser LPFGs thermal stability up to 600 °C. Furthermore, the temperature response presented in this work is higher compared to other high-temperature sensors based on LPFG fabricated in SMF 28 fiber [3,24].

#### 4.2.2. Turn Around Point Long-Period Fiber Gratings

**Figure 8:**
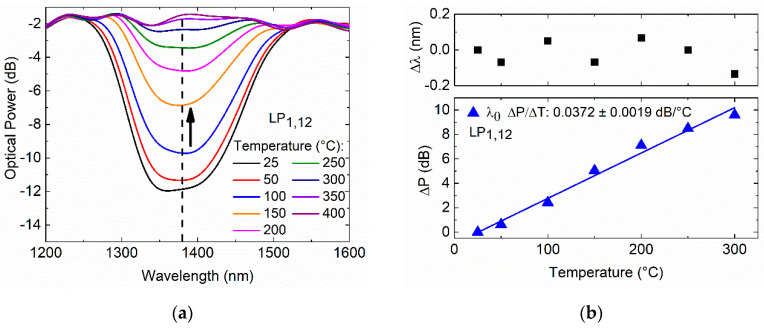
Fs-laser TAP LPFG inscribed with a period of 182.7 µm: (**a**) transmission spectra at annealing temperature; (**b**) normalized wavelength shift (■) and optical power variation (▲) as a function of the annealing temperature.

The thermal treatment of the 182.7 µm TAP LPFG was performed, and the transmission spectra response at the annealing temperatures was recorded, as shown in Figure 8.

From 25 to 300 °C, the TAP LPFG exhibits a single resonance that is wavelength-independent of temperature, as shown in Figure 8a. Furthermore, it is possible to see that the resonance optical power attenuation decreases (i.e., the coupling strength decreases) with the annealing temperature, leading to the resonance vanishing above the 300 °C. Figure 8b shows a temperature dependence (normalized to the room temperature, 25 °C) of 0.0372 ± 0.0019 dB/°C with linearity of $R^{2}\;>$ 0.98.

**Figure 9:**
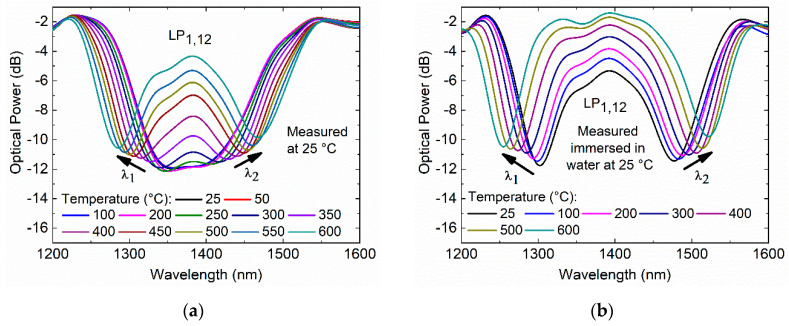
Fs-laser TAP LPFG inscribed with a period of 182.7 µm: (**a**) transmission spectra at room temperature; (**b**) transmission spectra immersed in water at room temperature.

As the annealing temperature increased, the TAP LPFG transmission spectra response was also recorded in air and water at room temperature, as presented in Figure 9a,b, respectively.

**Figure 10:**
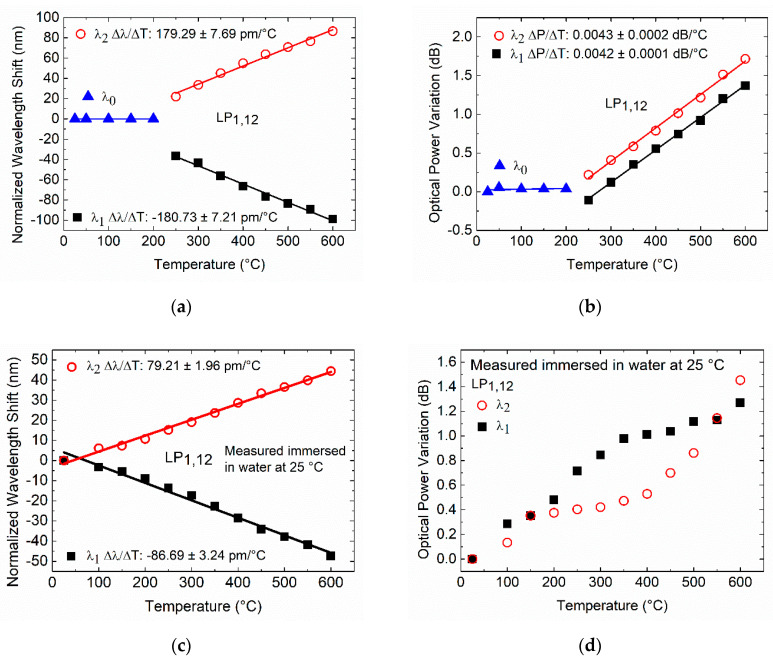
Fs laser 182.7 µm TAP LPFG: (**a**) normalized wavelength shift, and (**b**) normalized optical power variation in air at room temperature as a function of the annealing temperature; (**c**) normalized wavelength shift, and (**d**) normalized optical power variation in water at room temperature, as a function of the annealing temperature.

Figure 9a shows the behavior of the TAP LPFG in air at room temperature where, for annealing temperatures higher than 250 °C, it is possible to see that the resonance peak bifurcates and separates into two resonances. The temperature dependence of the TAP LPFG immersed in water leads to the appearance of two separated peaks, as shown in Figure 9b. Figure 10 shows the normalized wavelength shift and optical power variation measured of the TAP LPFG in air and water at room temperature, after a dwell time of 5 min at each annealing temperature.

In the temperature range from 25 to 200 °C, the TAP LPFG in air exhibits a single resonance that is wavelength-independent of temperature, as shown in Figure 10a. At 250 °C, the resonance has bifurcated and presents two peaks separated by 58.67 nm (blue-shift −36.62 nm, and red-shift +22.05 nm normalized to the room temperature, 25 °C). The two resonances continue to separate with increasing temperature, reaching a separation of 185.47 nm (blue-shift −98.89 nm, and red-shift +86.58 nm) in air (25 °C) after annealing at 600 °C. The TAP LPFG in air after the annealing presents a temperature dependence of −180.73 ± 7.21 pm/°C, and 179.29 ± 7.69 pm/°C, with linearity $R^{2}\;>$ 0.99 and, $R^{2}\;>$ 0.99 for the resonances *λ*_1_ and *λ*_2_, respectively. In Figure 10b, the normalized optical power of the TAP LPFG measured in air (25 °C) after the annealing became essentially constant in the temperature range from 25 to 200 °C. Above 200 °C, the TAP LPFG normalized optical power reaches a temperature dependence (normalized to room temperature, 25 °C) of 0.0042 ± 0.0001 dB/°C, and 0.0043 ± 0.0002 dB/°C, with linearities of $R^{2}\;>$ 0.99 and $R^{2}>$ 0.99 for the resonances *λ*_1_ and *λ*_2_, respectively. These results demonstrate that the annealing changes the RI modifications inscribed with the fs laser inside the core of the SMF-28e, as explained above for the cladding mode LP_1,6_ of a 372.5 µm LPFG. For the LP_1,12_ cladding mode with a TAP in its phase-matching curve for a wavelength around 1380 nm, the small changes in the coupling conditions caused by the annealing at temperatures higher than 200 °C split the resonance into two separate resonances, with positive and negative linear dispersions. The operation at the TAP for the 182.7 µm LPFG, with the performed thermal treatment, is ensured up to a temperature of 200 °C. To fabricate a TAP LPFG that is thermally stable at higher temperatures, it is necessary to increase the period of fabrication to values higher than 182.7 µm. Thus, with the performed thermal treatment through short-term thermal exposure (5 min for a given temperature), it is possible to operate around the TAP region with the 182.7 µm TAP LPFG fabricated by the fs laser.

Regarding the TAP LPFG immersed in water at 25 °C, the resonance presents two peaks separated by 175.15 nm (blue-shift −81.24 nm, and red-shift +93.91 nm normalized to the room temperature, 25 °C). As the annealing temperature increase, the two resonances continue to separate, reaching a separation of 266.84 nm (blue-shift −128.48 nm, and red-shift +138.36) in the water at room temperature after an annealing temperature of 600 °C. As shown in Figure 10c, temperature dependences of –86.69 ± 3.24 pm/°C, and 79.21 ± 1.96 pm/°C, with linearity $R^{2}\;>$ 0.98 and, $R^{2\;}\;>$ 0.99 were achieved for the *λ*_1_ and *λ*_2_ resonances, respectively. Figure 10d shows that the optical power variation, normalized to the room temperature, increases non-linearly. This behavior is related to the waveguide dispersion, which depends upon the order (LP_1,12_) of the cladding mode and the RI of the medium surrounding the cladding.

**Figure 11:**
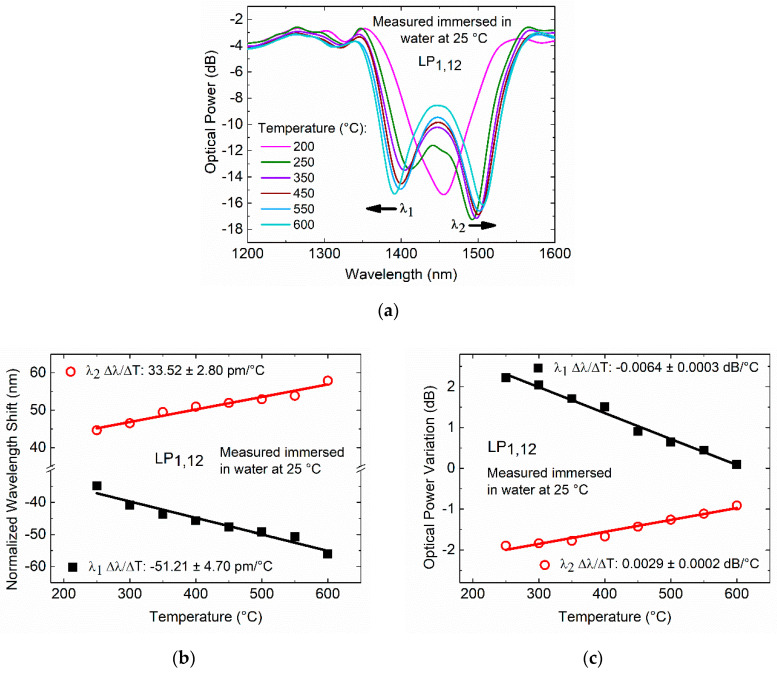
Fs laser TAP LPFG inscribed with a period of 192.5 µm measured in water at 25 °C: (**a**) transmission spectra after annealing at different temperatures, (**b**) normalized wavelength shift, and (**c**) normalized optical power variation.

The thermal treatment of the 192.5 µm period LPFG operating at the TAP in water was also performed, and the transmission spectra response were recorded, as shown in Figure 11a.

As fabricated, this TAP LPFG exhibits a single resonance at approximately 1448 nm. For annealing temperatures higher than 200 °C, it is possible to see that the resonance peak bifurcates and separates into two resonances, reaching a separation of 113.90 nm (blue-shift −56.01 nm and red-shift +57.89 nm) in water at room temperature after an annealing temperature of 600 °C. As shown in Figure 11b, temperature dependences of −51.21 ± 4.70 pm/°C and 33.52 ± 2.80 pm/°C, with linearity >0.94 and $R^{2}\;>$ 0.95 were achieved for the *λ*_1_ and *λ*_2_ resonances, respectively. Figure 11c shows that above 200 °C, the TAP LPFG normalized optical power reaches a temperature dependence (normalized to 200 °C) of 0.0064 ± 0.0003 dB/°C and 0.0029 ± 0.0002 dB/°C, with linearity $R^{2\;}>$ 0.98 and $R^{2\;}>$ 0.96 for the resonances *λ*_1_ and *λ*_2_, respectively.

When comparing the annealing temperature results of the LPFGs with 372.5 and 182.7 µm, the highest temperature dependence was seen when the LPFG was chosen to operate near the TAP in air (182.7 µm period). The temperature dependence of the LPFGs relies on the material and waveguide contributions. For the LPFG with a period of 372.5 µm, the material effect dominates, which is related to the change in the differential RI of the core and cladding arising from the thermo-optic effect. The LPFGs with periods of 182.7 and 192.5 µm present a lower material contribution to the temperature dependence. In this case, the waveguide contribution, resulting from changes in the LPFG period, dominates. Thus, the choice of the LPFG period is important to balance the two contributions to the temperature sensitivity and achieve a temperature-independent attenuation band and also to produce attenuation bands with temperature sensitivities (positive or negative) appropriate to specific applications. It was also verified that the spectral widths of the resonance peaks are broad when they are close to the turning points, and narrower when they are further away from them. The performed thermal treatment through short-term thermal exposure enables an optimization of the fabrication of the LPFGs, mainly at the TAP region (higher sensitivities). The LPFG period can be tuned through the annealing for a short time and also reach thermal stability up to the utilized annealing temperature. Furthermore, the LPFGs annealing procedure made before LPFGs functionalization with nano-assembled thin films is essential to ensure that the LPFG resonance wavelength and the intensity remain unchanged with the temperatures used in the deposition procedures [7].

## 5. Conclusions

The fabrication of LPFGs in an SMF-28e fiber through fs laser direct writing was demonstrated. The non-linear nature of the absorption process of the fs laser pulses, combined with the high precision and flexibility of the developed direct writing system allowed the fabrication of LPFGs with different cladding order modes (e.g., LP_1,6_ and LP_1,12_) by just selecting a particular period (e.g., 372.5 and 182.7 µm). The LPFGs operating at the TAP in air and water were fabricated with an RI modulation period of 182.7 and 192.5 µm, yielding a resonance wavelength at approximately 1380 and 1448 nm, respectively. The experimental evidence shows that the fabrication of LPFG at the TAP requires a precise control on the fiber alignment, fiber tension during fabrication, and submicrometric control of the grating period.

The experimental results show that the fabricated LPFGs must be annealed to stabilize their spectra. We demonstrated the fine-tuning of the resonance band’s position and thermal stability up to 600 °C. The fabrication of LPFGs at the TAP, with thermal stability up to 200 °C was achieved, which holds on the high sensitivity characteristics. From 25 to 300 °C, the TAP LPFG exhibits a single resonance that is wavelength-independent of temperature and presents a temperature dependence of 0.0372 dB/°C. Furthermore, temperature dependences of −180.73 and 179.29 pm/°C were achieved for the *λ*_1_ and *λ*_2_ resonances, respectively, in the temperature range from 250 to 600 °C. The temperature dependence of 124.76 pm/°C up to 600 °C was achieved for the 372.5 µm LPFG. Thus, it was demonstrated that an LPFG has attenuation bands with positive, negative, and independent wavelength responses to temperature. The temperature dependence is associated with the LPFG period, the order of the cladding mode, and the composition of the optical fiber.

The robust nature of the fabricated devices opens the possibility of high-temperature sensing with high sensitivity and also chemical and bio-chemical sensing with a thermally stable and temperature-independent attenuation band.

## Figures and Tables

**Table 1:** Comparison between two pairs of LPFGs.

LPFGs Number	Mode	Λ (µm)	*λ*_res_ (nm)	Intensity (dB)	FWHM (nm)
1	LP_1,6_	372.5	1494.97	−11.86	11.97
2			1495.04	−11.71	12.63
3	LP_1,12_	182.7	1385.45	−9.76	64.54
4			1385.43	−11.17	65.55
